# Q‐Switched 785 nm laser in the treatment of labial melanotic macules

**DOI:** 10.1111/srt.13581

**Published:** 2024-01-14

**Authors:** Giustino Gallo, Shady Mahmoud Ibrahim, Beatrice Marina Pennati, Laura Pieri, Tiziano Zingoni, Paolo Bonan

**Affiliations:** ^1^ El.En. Group Florence Italy; ^2^ Dermatology and Venereology Faculty of Medicine Al‐Azhar University Cairo Egypt; ^3^ Laser Cutaneous Cosmetic & Plastic Surgery Unit Villa Donatello Clinic Florence Italy

Dear Editor,

1

Physiological lip melanosis is a disorder with a multifactorial aetiology. It varies with ethnicity and is typical of people with darker skin tones. Benign conditions (ephelides, lentigines, labial melanotic macule), malignant instances (pigmented squamous cell carcinoma, malignant melanoma), drug‐induced, post‐inflammatory hyperpigmentation (PIH), endocrine disorders, heavy metals, smoking, amalgam tattoo, Laugier‐Hunziker syndrome and lentiginosis syndromes (Peutz–Jegher's syndrome) are among the factors that can cause lip pigmentation labial melanotic macules are pigmented benign macules with a linear increase in pigment in the basal layer without any melanocyte proliferation and without any vascular component.^1^

Although treatment is not needed, it is requested by many patients, who are greatly distressed by this condition. For lip melanosis, there is, unfortunately, no effective treatment.^2^ Patients frequently experiment with whitening creams, camouflage lipsticks and lip tattoos without getting the desired outcome.^3^

Histological evidence of lip darkening shows abnormal basal layer melanin granule aggregation with a regular number of melanocytes and an excess of dermal melanophages.^4^ These are good targets for Q‐switched (QS) lasers because they contain melanin which is the interested chromophore. This way, collateral tissue damage can be reduced. It has been demonstrated that the QS 532‐nm laser works perfectly to treat hyperpigmented lips. Nevertheless, negative effects are more common, last longer and are more intense than with the QS 1064‐nm laser.^5^ The QS picosecond lasers emit pulses that are much shorter than the target's thermal relaxation time so they raise the peak temperature without inflicting thermal damage on the nearby tissues.

Given this, this study aimed to conduct a preliminary assessment of the application of a picosecond QS 785 nm handpiece (El.En Group, Florence, Italy) for the treatment of lip hyperpigmentation, to minimize blood absorption and maximize the absorption of melanin.

A 51‐year‐old male patient with lower lip melanosis is the subject of this case study. The parameters for treatment were spot size 3 mm, fluence 2.8 J/cm^2^, frequency 2 Hz and single pass modality. To get the most out of the technology, a contact sensor was included on the device. The last assessment and follow‐up appointments were conducted 24 h and 14 days following the last laser treatment.

Our findings indicate that the patient's mucosa has very quickly almost completely recovered, when compared to the skin (see Figure 1).

**FIGURE 1:**
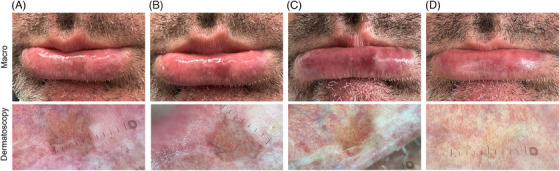
The clinical progress of the lower lip melanosis case is shown. (A) At baseline; (B) Right after treatment; (C) Follow‐up at 24 h after treatment, and (D) Follow‐up at 30 days after treatment. An almost complete resolution is visible.

Additionally, the patient was given a visual analogue scale (VAS) with a range of 1 to 10 and the value he reported indicated a high degree (VAS = 9) of satisfaction with complete disappearance of the lip hyperpigmentation.

According to Loh et al. (2021), 785 nm may be the preferred wavelength for treating darker lesions and darker skin types. On the other hand, the 730 nm emitting devices may be preferable for treating lightly pigmented lesions because the melanin absorption coefficient at 730 nm is expected to be 30% higher than at 785 nm.^6^

Furthermore, due to melanin's preferred absorption over haemoglobin, the 785 nm wavelength may be better compared to 1064 and 532 nm when targeting skin or lip pigment.^7^

Indeed, we treated a different and unusual body district such as the internal part of the lower lip. It has unique characteristics being a mucosa and a vascular component‐rich area. Of course, different laser strategies could have been used to target melanin, such as the 675 nm wavelength devices or the 532/1064 nm lasers, that we previously used in other investigations.^8^ Nevertheless, in the clinical case presented we decided to have a conservative approach during the treatment, to focus on the melanotic component only and avoid the vascular one. This way, no side effects were registered, and the treatment was almost pain‐free and bearable by the patient.

Oral mucosa healing happens way faster than skin healing. Indeed, networks related to wounds of the oral mucosa regulate inflammatory responses and epithelial cell differentiation.^9^

In summary, the use of the 785 nm wavelength in our study was warranted to improve melanin absorption when compared to blood absorption. It has been confirmed that the device utilized in this study has a good safety record. The nearly complete elimination of lip hyperpigmentation and the ability to resume normal activities as soon as the laser sessions concluded contributed to the patient's high level of satisfaction following the treatment. The technology also had the benefit of not having any negative long‐term side effects or pain.

## Data Availability

The data that support the findings of this study are available on request from the corresponding author. The data are not publicly available due to privacy or ethical restrictions.
